# Stability of adrenocorticotropic hormone in whole blood samples: effects of storage conditions

**DOI:** 10.11613/BM.2024.030702

**Published:** 2024-08-05

**Authors:** François Fraissinet, Hélène Girot, André Gillibert, Anaïs Melin, Julie Fettig, Valéry Brunel

**Affiliations:** 1 General Biochemistry Department, Institute for Clinical Biology, Rouen University Hospital, Rouen, France; 2 UNIROUEN, Normandie University, INSERM, U1239, NorDiC, Rouen, France; 3 Biostatistics Department, Rouen University Hospital, Rouen, France

**Keywords:** adrenocorticotropic hormone, hemolysis, preanalytical phase, specimen stability, whole blood

## Abstract

**Introduction:**

Adrenocorticotropic hormone (ACTH) is a peptide secreted by pituitary gland that plays an important role in regulating cortisol secretion. Its determination is difficult because of instability in whole blood. Several factors that influence ACTH stability in blood before analysis have been identified: temperature, hemolysis, time to centrifugation and presence of protease inhibitors. Published results on ACTH whole blood stability seem contradictory.

**Materials and methods:**

We performed a stability study in 10 healthy volunteers. Three different conditions were tested: ethylenediaminetetraacetic acid (EDTA) at 4 °C, EDTA + aprotinin at 4 °C, EDTA + aprotinin at room temperature. Stability was evaluated for 8 hours. Adrenocorticotropic hormone measurements and hemolysis index were performed respectively on Cobas e602 and c701 (Roche Diagnostics, Mannheim, Germany). We compared percentage deviations with total change limit using a threshold of 7.5%.

**Results:**

We showed that ACTH is stable 8 hours with EDTA at 4 °C, 4 hours with EDTA + aprotinin at 4 °C and 2 hours with EDTA + aprotinin at 22 °C.

**Conclusions:**

Aprotinin does not appear to give ACTH greater stability but can be used without exceeding 4 hours at 4 °C. Refrigerated pouch transport also seems to be more appropriate for ACTH in whole blood.

## Graphical abstract

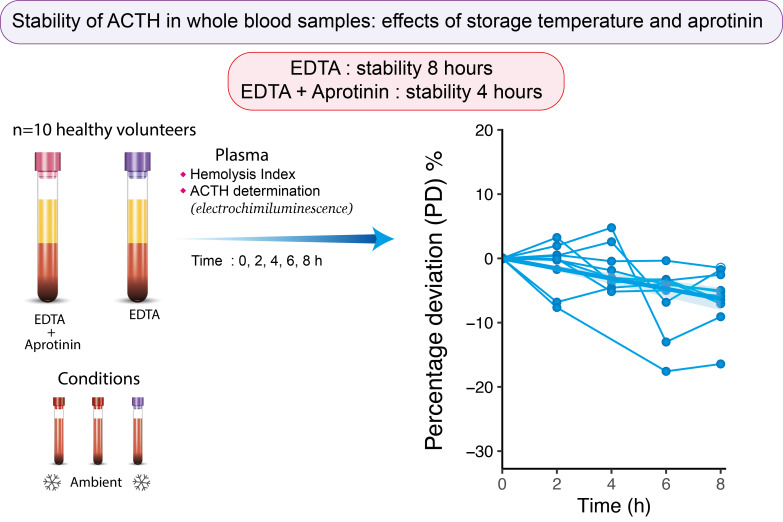

## Highlights

• Adrenocorticotropic hormone (ACTH) is a peptide sensitive to proteolytic degradation in the blood

• The stability of ACTH in the blood depends on various factors: temperature, hemolysis, time to centrifugation and presence of protease inhibitors

• ACTH is stable in whole blood 8 hours with EDTA and 4 hours with EDTA and aprotinin

• Transport at 4 °C seems most appropriate to prevent ACTH degradation

## Introduction

Adrenocorticotropic hormone (ACTH) is a 39 amino-acid peptide derived from the cleavage of a glycoprotein, the pro-opiomelanocortin. Measurement of ACTH is necessary in the exploration of Cushing syndrome, adrenal insufficiency and hypothalamic-pituitary disorders (*1*). In whole blood sample, ACTH is unstable due to proteolytic degradation. The resulting preanalytical recommendations are to centrifuge and freeze the sample immediately or within one hour by keeping it refrigerated. In routine practice, ethylenediaminetetraacetic acid (EDTA)-containing samples are recommended to protect against protein changes, notably due to calcium dependent peptidase (*2*). Christensen *et al.*, demonstrated that EDTA samples could be stored at room temperature for four hours allowing the removal of the refrigerated step (*3*). Other authors investigated the protective role of aprotinin, a protease inhibitor, on ACTH stability in whole blood. These authors suggested that whole blood storage in EDTA tubes containing aprotinin at room temperature was acceptable for up to 4 hours and was better than in EDTA tubes containing aprotinin in refrigerated condition (*4*). However, these contradictory results of stability in whole blood were not studied beyond 4 hours in these studies. We perform approximately 2000 ACTH assays *per* year, most of which are for the endocrinology department which is not located on the central site. Hence, the manufacturer’s recommendations are to use chilling K_2_-EDTA or K_3_-EDTA tubes, to transport samples on ice, centrifuge quickly at 4 °C and after freezing of the samples to carry out the dosages in series. The manufacturer does not mention sample tubes containing aprotinin. In our center, refrigerated sample transport is not done in water with ice but in refrigeration systems which maintain the temperature at 4 °C. The aim of this study was to investigate whether the addition of aprotinin to the sample was suitable for ACTH measurement and which storage method was the most advisable for ACTH. The influence of hemolysis on ACTH concentrations was assessed for each condition by measuring the hemolysis index.

## Materials and methods

**Figure 1:**
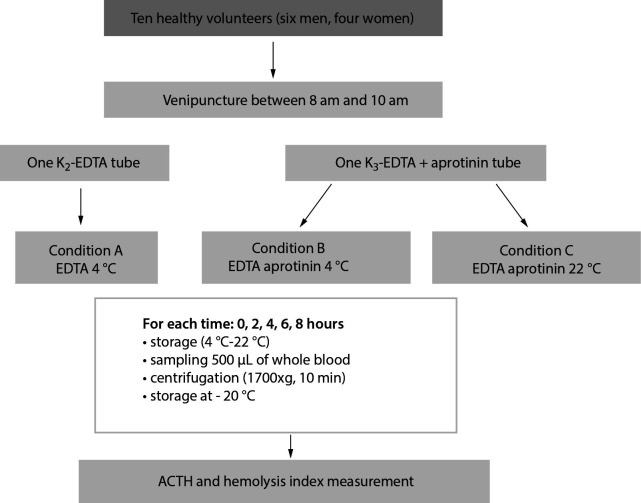
Flow-chart of the study. ACTH - adrenocorticotropic hormone. EDTA - ethylenediaminetetraacetic acid.

After informed consent was obtained, venous blood was collected between 8 am and 10 am from 10 healthy volunteers (six men, four women) with the device BD Vacutainer UltraTouch Push Button with 21G needle (Becton Dickinson, Franklin Lakes, USA). Informed consent was obtained from all individuals included in this study. The exclusion criterion concerned patients with insufficient venous capital. Research complied with all relevant national regulations, and institutional policies and was conducted in accordance with the Helsinki Declaration (Number clinical trials: NCT04266587). The clinical study began with samples from all healthy volunteers on 18/11/2021 and ended on 2/12/2021 with all dosages. The flow-chart of this clinical study is shown in Figure 1. Each volunteer provided two samples, comprising one K_2_-EDTA sample and one K_3_-EDTA + aprotinin samples (Becton, Dickinson and Company, Franklin Lakes, NJ, USA). Three conditions were tested; condition A: K_2_-EDTA samples at 4 °C; condition B: K_3_-EDTA + aprotinin at 4 °C; condition C: K_3_-EDTA + aprotinin at room temperature. The temperature intervals defined by the laboratory are respectively: 21.5 ± 3.5 °C and 5 ± 3 °C. K_3_-EDTA + aprotinin sample was split in two after mixing to test two conditions: K_3_-EDTA + aprotinin at 4 °C and K_3_-EDTA + aprotinin at 22 °C. At each storage time (T0, T2, T4, T6 and T8 h), 500 μL of whole blood were drawn from each condition, samples were mixed one by one by successive turns, then were centrifuged (1700xg, 10 min, 4 °C) and plasma aliquots were stored frozen at - 20 °C until analysis. After thawing, plasma samples were analyzed in single (no replicates) in the next 15 minutes and hemolysis index was determined on Roche instrument, an index of 1 corresponding to 10 mg/dL of cell free hemoglobin. All assays were performed after thawing of samples with the same lot of reagents and in one batch. Results of ACTH are expressed in pg/mL. ACTH assays were performed on a Roche cobas e602 analyzer (Roche Diagnostics, Mannheim, Germany) and followed the method provided by the manufacturer. Hemolysis was assessed using hemolysis index (H-index) on Roche cobas c701 analyzer (Roche Diagnostics, Mannheim, Germany). For each storage time and each condition, percentage deviation and instability equations were calculated according to guidelines using the formula: (ACTH Tx - ACTH T0) / ACTH T0 (*5*). The mean of percentage deviation of each condition was compared with a total change limit (TCL) of 7.5% previously defined: TCL = ((2.77 x analytical imprecision)^2^ + (0.5 x within subject variation)^2^)^1/2^ (*6*). This value also corresponds to twice our analytical interassay coefficient of variation (CV = 3.7%). Existence of outliers was verified according to the Grubbs test. Hemolysis index and percentage deviations were compared by repeated measures two-way ANOVA, both using Tukey’s test for multiple comparisons. Statistical analysis were performed with R software (4.1.3) and GraphPad Prism 8.0 (GraphPad Software, Boston, USA).

## Results

**Figure 2:**
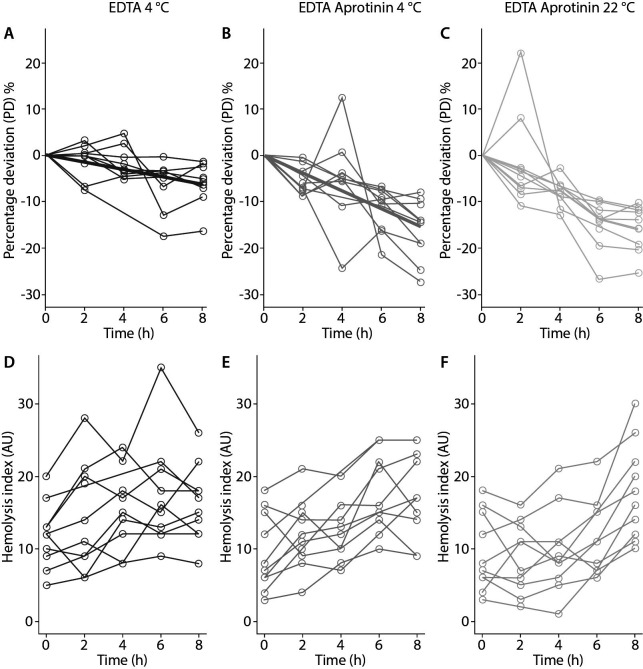
Stability of ACTH in whole blood with different conditions. Percentage deviations and instability equations of ACTH concentrations (A, B, C) and hemolysis index (D, E, F) for each condition tested: EDTA 4 °C, EDTA + aprotinin 4 °C, EDTA + aprotinin 22 °C. ACTH - adrenocorticotropic hormone. EDTA - ethylenediaminetetraacetic acid.

An outlier was highlighted concerning the percentage deviation. It corresponds to a low ACTH concentration of 3.39 pg/mL at T4. This statistically outlier has been removed for subsequent statistical tests. For hemolysis index, two samples were identified as outliers and presented a hemolysis index higher than 30. Percentage deviations and hemolysis index of the different storage times of the three conditions are presented in Figure 2. For condition A (EDTA 4 °C), percentage deviation does not exceed TCL for any time; for condition B (EDTA + aprotinin 4 °C), it exceeded TCL at 6 and 8 hours and for condition C (EDTA + aprotinin 22 °C) at 4, 6 and 8 hours (Table 1).

**Table 1:** Mean of percentage deviation and instability equation for each time and condition for adrenocorticotropic hormone measurements

**Time (h)**	**Condition A** **EDTA 4 °C**	**Condition B** **EDTA + aprotinin 4 °C**	**Condition C** **EDTA + aprotinin 22 °C**
2	- 1.06 (- 7.96; 5.84)	- 5.46 (- 11.10; 0.18)	- 1.64 (- 20.85; 17.56)
4	- 1.84 (- 8.23; 4.96)	- 5.23 (- 24.25; 13.78)	- 8.00 (- 13.59; - 2.42)
6	- 6.10 (- 16.33; 4.12)	- 11.68 (- 21.15; - 2.21)	- 14.95 (- 24.81; - 5.10)
8	- 6.09 (- 14.65; 2.47)	- 16.18 (- 28.80; - 3.56)	- 15.73 (- 25.19; - 6.27)
Instability Equation	PD = - 0.783 x time (h), R^2^ = 0.928 P = 0.001	PD = - 1.928 x time (h), R^2^ = 0.975 P < 0.001	PD = - 2.0907 x time (h), R^2^ = 0.969 P < 0.001
Percentage deviation were expressed as mean and 95% confidence interval. Percentage deviation were calculated with formula (ACTH Tx – ACTH T0)/ACTH T0 for each condition. ACTH - adrenocorticotropic hormone. EDTA - ethylenediaminetetraacetic acid.			

The conditions were compared using an ANOVA test with repeated measures. For percentage deviation of ACTH, a significant influence of the condition is observed: F (1.436, 12.93) = 5.510, P = 0.026 with comparisons par pairs showing (EDTA 4 °C *vs* EDTA + aprotinin 4 °C, P = 0.028; EDTA 4 °C *vs* EDTA + aprotinin 22 °C, P = 0.011, EDTA + aprotinin 4 °C *vs* EDTA + aprotinin 4 °C, P = 0.966). Similarly, for index hemolysis, a significant influence of the condition is observed: F (1.157, 10.41) = 9.686, P = 0.009 with comparisons par pairs showing (EDTA 4 °C *vs* EDTA + aprotinin 4 °C, P = 0.337; EDTA 4 °C *vs* EDTA + aprotinin 22 °C, P < 0.001; EDTA + aprotinin 4 °C *vs* EDTA + aprotinin 4 °C, P < 0.001).

## Discussion

Our study confirms the effects of several factors on ACTH stability: tube containing aprotinin, delivery time, temperature and hemolysis. Aprotinin is a serine protease inhibitor which prevents proteolytic degradation of peptides. In fact, blood contains mainly numerous proteases: serine proteases (coagulation factors), cysteine proteases (cathepsins) and metalloproteases (*7*). Ethylenediaminetetraacetic acid as ion chelator inhibits proteolysis by metalloproteases or metal-ion dependent proteases. The effects of aprotinin were studied on plasma for 72 h and the condition EDTA + aprotinin 4 °C was found the most stable (*8*). Our findings are consistent with these results, in a whole blood matrix even if in our case, EDTA alone provides the best stability. However, others reported conflicting results which showed better stability of ACTH in the aprotinin condition at room temperature (*4*). It is also important to quickly separate the plasma from red blood cells. The time to analysis must also be as short as possible, we chose 15 minutes as others (*9*). Storage temperature also plays an important role in ACTH stability. We found that ACTH was more stable in EDTA whole blood with aprotinin at 4 °C (4 hours) than without aprotinin at room temperature (2 hours). We can suppose that storage at 4 °C inhibits proteolytic enzyme activity resulting in the improvement of ACTH stability. These enzymes can be inhibited by protease inhibitors as aprotinin or by low temperature (4 °C). We hypothesized that storage at 4 °C would inhibit the proteolytic enzyme activity which was not inhibited by aprotinin, both conditions resulting in the improvement of ACTH stability. However, hemolysis may increase at 4 °C because cooling may cause the rupture of erythrocyte membrane cells. A little hemolysis, even invisible to the eye (10-20 mg/dL of hemoglobin) leads to a decrease in ACTH concentrations which could explain the different results between studies (*9*). Indeed, we observed a difference between 0 and 8 hours but less hemolysis (two samples with a visible hemolysis) compared to a previous study (*4*). These authors used an ice water mixture to store tubes. From experience, we know that this type of storage is more hemolyzing than storage in a refrigerated chamber at 4 °C. It should also be noted that we no longer use storage in ice water in our routine practice and prefer thermostated pouch systems, which induce less hemolysis. In addition, the use of pre-chilled tubes does not seem to be necessary (*10*). To improve ACTH stability, the addition of N-phenyl maleimide, a cysteine protease inhibitor was also studied by others, showing that this molecule stabilizes ACTH in plasma, but is not used commonly because it induces major hemolysis in whole blood (*9*). The effect of EDTA amount on ACTH concentration has also been studied, and increased EDTA concentration results in a significant decrease in ACTH concentration (*11*). In our study, ACTH concentrations at T0 were slightly higher with aprotinin tube than EDTA even if the difference is not significant. The higher ACTH concentration in the aprotinin tube could be explained by an effect of aprotinin at early times which then decreases over time. The same amount of EDTA is present in K_2_-EDTA and in K_3_-EDTA 4 mL tubes (7.2 mg - 1.8 g/L), and a higher amount in K_3_-EDTA 5 mL aprotinin tube (8.1 mg - 1.62 g/L) which could explain the difference. In addition, we made sure to fill the tubes correctly at the time of sampling. It should also be noted a greater but not significant hemolysis of the EDTA tube compared to the aprotinin tube. Recently, it has been shown that freeze-thaw cycles can decrease ACTH concentrations which was not highlighted in previous studies (*12*). In our study, the samples did go through a cycle of freezing and thawing that could have altered the stability of ACTH.

This study has several limitations. Only 10 subjects were included and their ACTH concentrations are in reference range (7-63 pg/mL). It would be interesting to test high ACTH concentrations. Indeed, the condition EDTA at room temperature has not been tested. We compared percentage deviation with TCL, some authors use a threshold > 10% for which ACTH stability would be 8 hours for all three conditions. Nevertheless, the use of TCL seems more appropriate and takes into account analytical and biological variations (*11*).

In conclusion, in our study, acceptable times for ACTH preanalytical phase are 4 hours for EDTA + aprotinin when blood samples are stored at room temperature and 8 hours when stored at 4 °C. Each laboratory must keep in mind that the whole blood stability of ACTH is dependent on temperature, presence of protease inhibitor, time to centrifugation and hemolysis which may itself be dependent on the other parameters.

## Data Availability

The data generated and analyzed in the presented study are available from the corresponding author on request.
